# Supplementary figures and images for: Clade-D auxin response factors regulate auxin signaling and development in the moss Physcomitrium patens
Source: PLoS Biol. 2023 Jun 14;21(6):e3002163. doi: 10.1371/journal.pbio.3002163 (PMC10299833; doi:10.1371/journal.pbio.3002163)

A

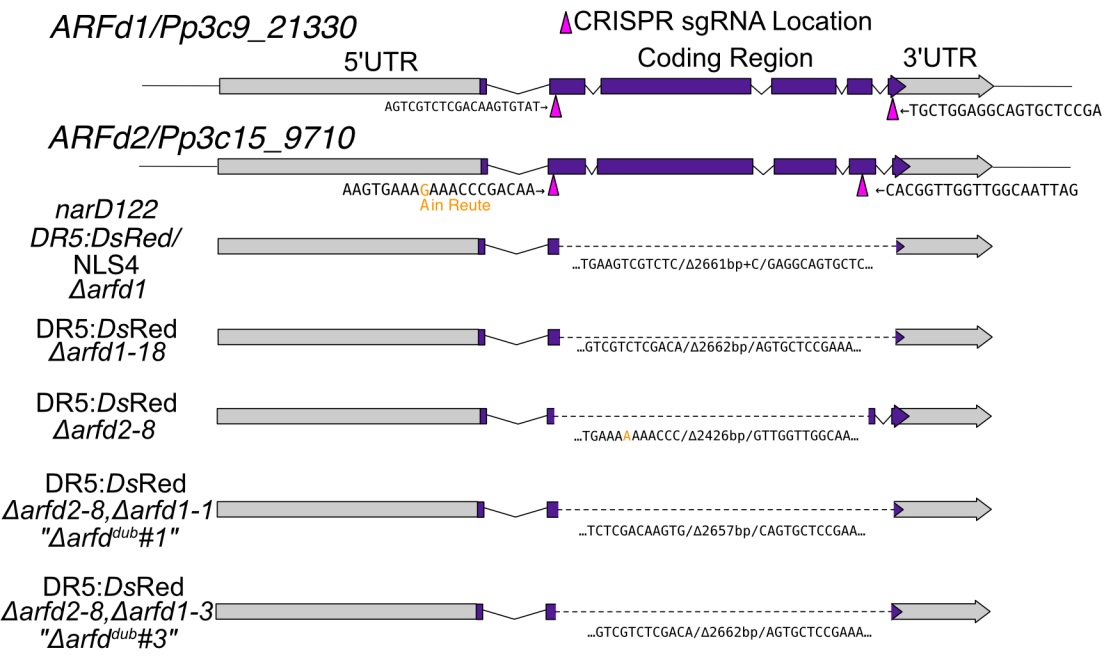

B

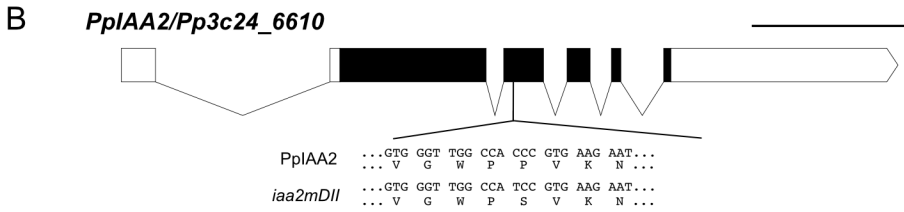

C

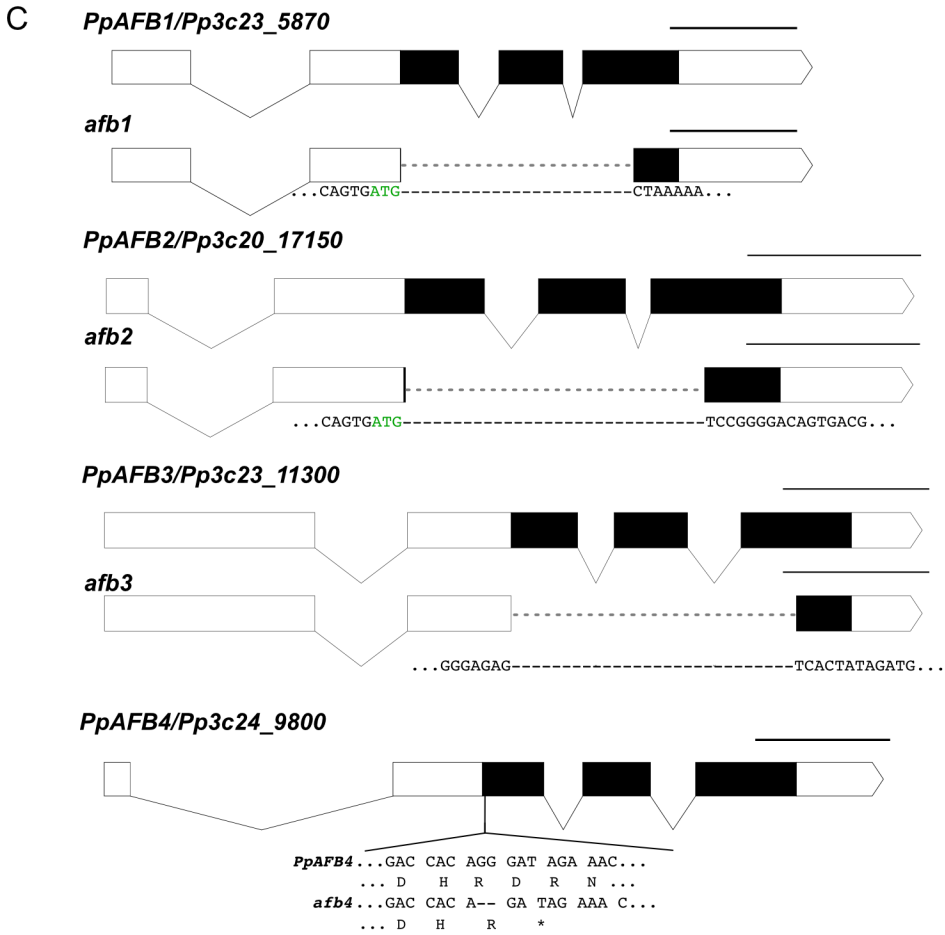

Supplement: S1 Fig — (A) Models of PpARFd1 and PpARFd2, including the targets of CRISPR/Cas9-mediated knock out. (B) Schematic of PpIAA2 and PpAFB mutations. (A) A single nucleotide mutation (red text) in the degron (DII) motif in PpIAA2 results in a stabilized auxin repressor. (C) Alleles recovered when generating afb1,2,3,4 line. Green text indicates endogenous start codon. Start codon was knocked out in PpAFB3. The 2 nt deletion in PpAFB4 generates a frameshift mutation, resulting in a premature stop codon. The afb4 peptide is 21 amino acids long. (PDF) [file pbio.3002163.s001.pdf]

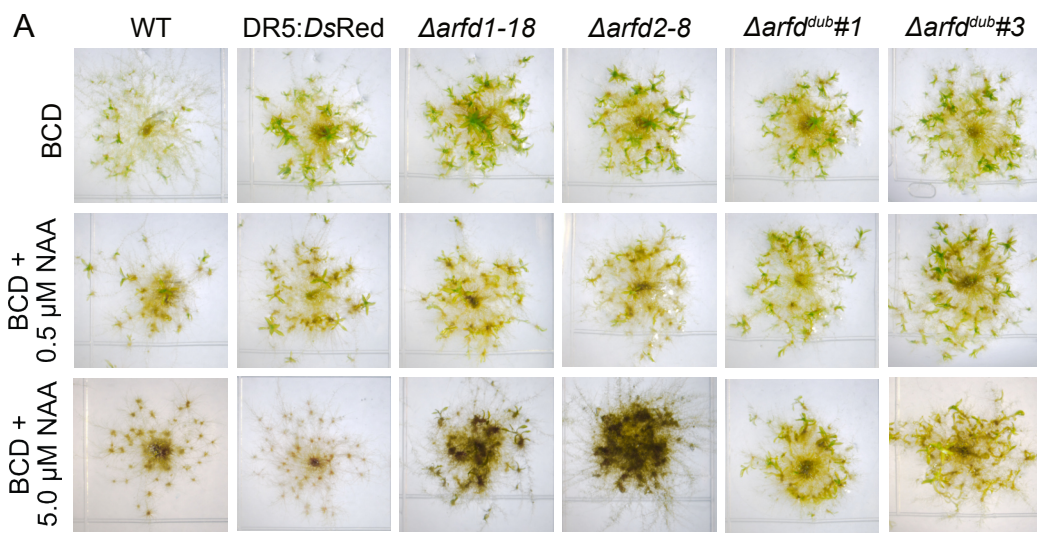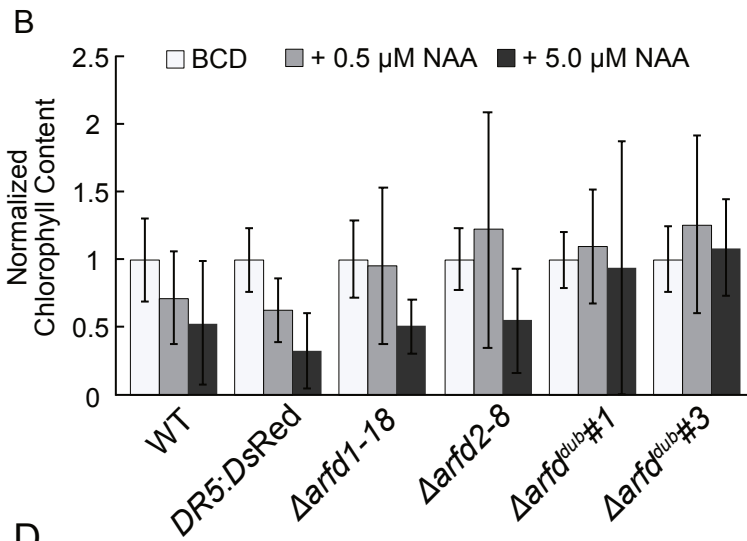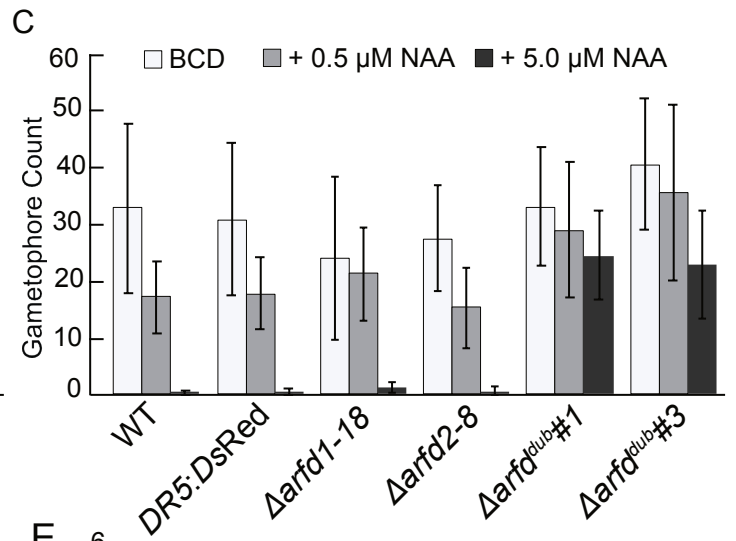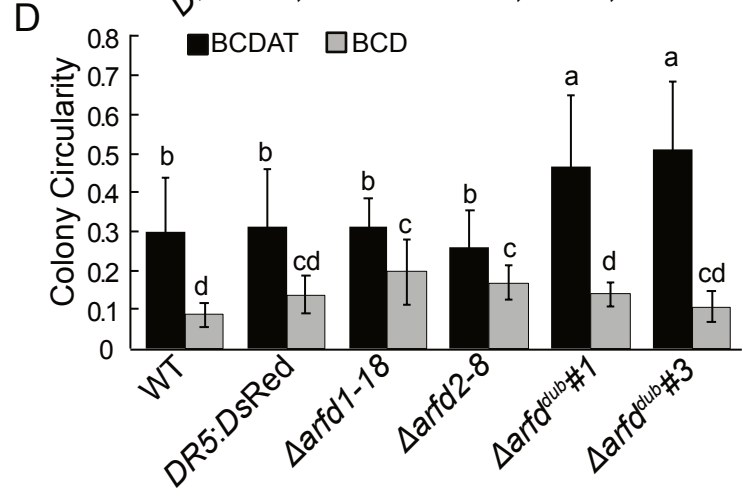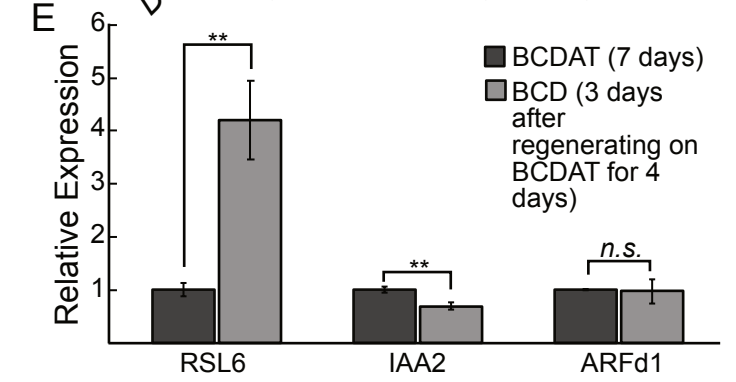

Supplement: S2 Fig — (A) Images of 21-day-old spot inocula on BCD medium. (B) Normalized chlorophyll content of 21-day-old colonies grown on BCD supplemented with the indicated concentration of NAA. (C) Gametophore counts of 21-day-old colonies grown on BCD supplemented with the indicated concentration of NAA. For both B and C, n ≥ 18 across 3 replicates. Error bars are standard deviation. (D) Morphology of 21-day-old colonies of arfd mutants are similar on low-nitrogen BCD media. On BCDAT, however, the arfddub are significantly more circular. Letters indicate statistical groups as determined by a TukeyHSD post hoc test of an ANOVA. (E) RSL6, an auxin-induced gene, is significantly up-regulated in tissue grown on BCD for 3 days. Expression level normalized to gene expression on tissue kept on BCDAT for 3 days. IAA2 is nominally down-regulated in repsonce to BCD treatment, while ARFd1 is not differentially expressed. Bar graphs indicate mean of 3 biological replicates, error bars are standard error of the mean. ** = p < 0.005. The underlying data for panels B, C, D, and E are in S1 Data. Representitive images for colony morphology image analaysis are in S2 Data. (PDF) [file pbio.3002163.s002.pdf]

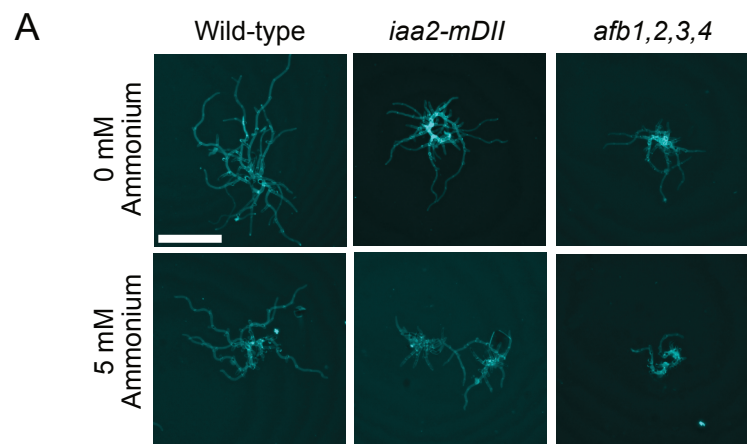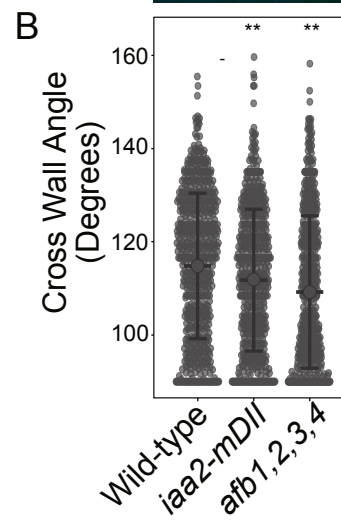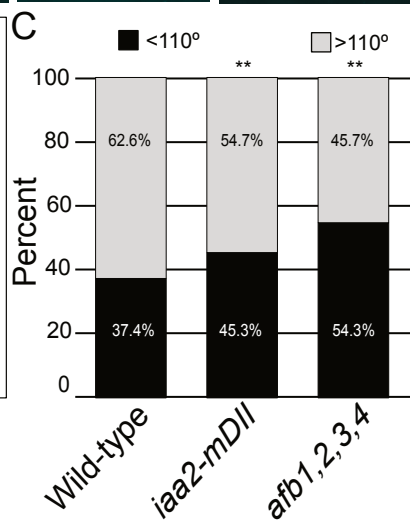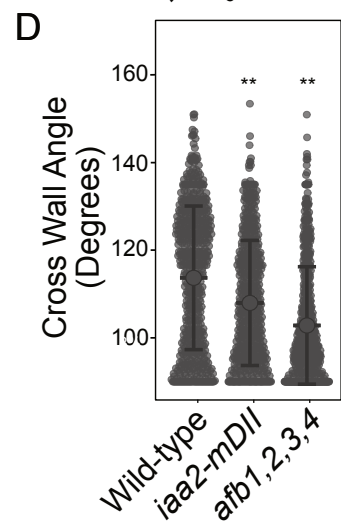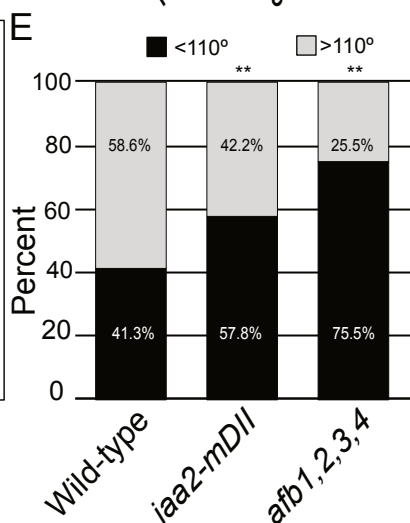

Supplement: S4 Fig — (A) Representative micrographs of 7-day-old plants regenerated from single protoplasts. WT, iaa2-mDII, and afb1,2,3,4 each exhibit changes in morphology in response to ammonium in the medium. Scale bar is 500 μm. (B) Cross-wall angles of protonemata from WT (n = 660), iaa2-mDII (n = 631), and afb1,2,3,4 (n = 587) plants across 3 biological replicates grown on BCD medium. Protonemata from both mutant lines have a smaller average cross-wall angle compared to wild type (Student’s T test, p < 0.001. Error bars are standard deviation.). (C) Protonemata form both lines are enriched in chloronemata (Kolmogorov–Smirnov test, p < 0.001). (D) Cross-wall angles of protonemata from WT (n = 583), iaa2-mDII (n = 583), and afb1,2,3,4 (n = 560) plants across 3 biological replicates grown on BCDAT medium (Student’s T test, p < 0.001. Error bars are standard deviation.). (E) Protonemata form both lines are enriched in chloronemata (Kolmogorov–Smirnov test, p < 0.001). The underlying data for panels B, C, D, and E are in S1 Data. Representitive images demonstrating cross-wall image analaysis are in S2 Data. (PDF) [file pbio.3002163.s004.pdf]

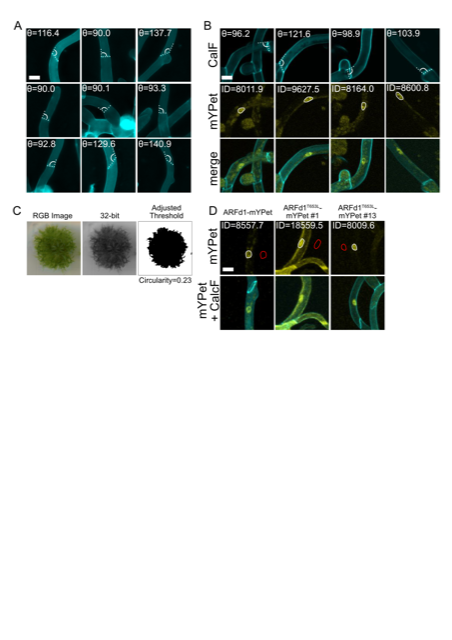

Supplement: S2 Data — (A) Representive cross-wall measurements. Scale bar is 5 μm. (B) Representive cross-wall measurements and Integrated Density (ID) quantification of ARFd1-mYPet signal in the nucleus (white outline). Scale bar is 5 μm. (C) Representitive example of imaging processing for colony morphology quantificiation. (D) Representitive micrographs of nuclear ARFd1-mYPet and ARFd1T653L-mYPet signal quantification. White outline is ROI for nucleus. Red outline is ROI for background subtraction. Only 1 background ROI was measured for each micrograph containing multiple nuclei. Scale bar is 5 μm. (TIFF) [file pbio.3002163.s007.tiff]
