# Supplementary material for: Clade-D auxin response factors regulate auxin signaling and development in the moss Physcomitrium patens
Source: PLoS Biol. 2023 Jun 14;21(6):e3002163. doi: 10.1371/journal.pbio.3002163 (PMC10299833; doi:10.1371/journal.pbio.3002163)
Supplement: S3 Fig — (A) Gene diagram of pARFd1:PpARFd1-mYPet locus with the PB1 domain in blue. Indicated location of sgRNAs (red text) used to create T653L and Y643* mutations. Scale bar is 1,000 nts. (B) Sequences of WT, narD122, mutating oligomer, and recovered arfd1T653L or arfd1Y643* mutants. Magenta text highlights codon change, with green highlighting silent mutations intended to introduce restriction enzyme sites. Red/pale red text indicates the protospacer-adjacent motif (PAM), while orange text highlights silent mutations introduced to abolish sgRNA binding. For arfd1T653L, only the PAM sequence needed to be changed. For arfd1Y643*, there were no available silent mutations for the lysine residue that would not also disrupt the intron-exon slice site. (C) Micrographs of ARFd1-mYPet and 2 independent lines of arfd1T653L-mYPet. Magenta is chloroplast autoflouresence, yellow is mYPet signal. Often, both ARFd1 and arfd1T653L formed single subnuclear focus (white arrow head) and scale bar is 5 μm. (B) mYPet signal, normalized to ARFd1-mYPet. Signal of arfd1T653L-mYPet (n ≥ 66 cells across 2 replicates) is roughly double that of ARFd1 (n = 170 cells across 2 replicates). * = p ≤ 0.001. The underlying data for panel D are in S1 Data. Representitive images describing nuclear signal image analaysis are in S2 Data. (PDF) [file pbio.3002163.s003.pdf]

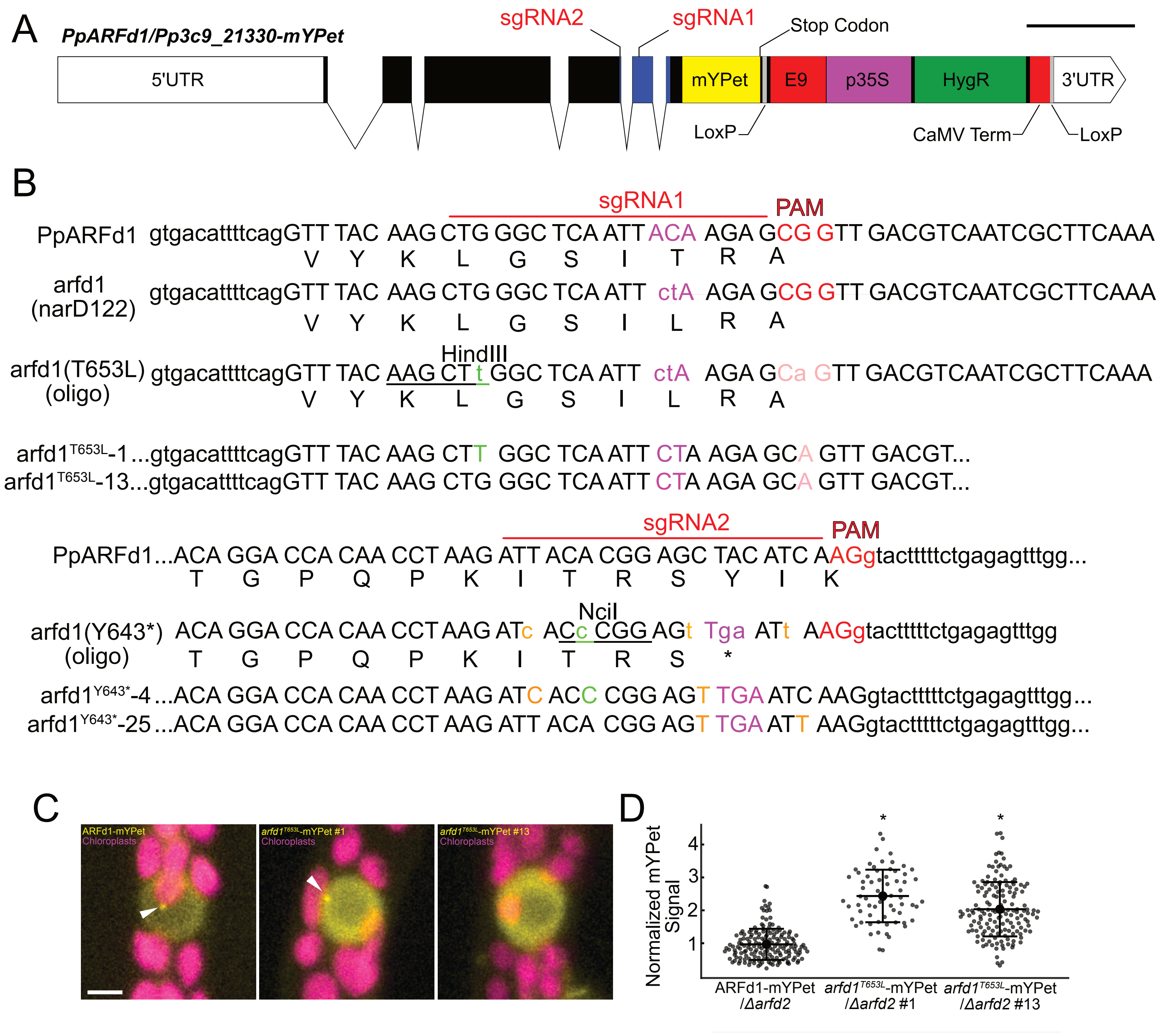
A
PpARFd1/Pp3c9_21330-mYPet
sgRNA2
sgRNA1
Stop Codon
5'UTR
mYPet
E9
p35S
HygR
3'UTR
LoxP
CaMV Term
LoxP
B
sgRNA1
PAM
PpARFd1 gtgacattttcagGTT TAC AAG CTG GGC TCA ATT ACA AGA GCG GTT GACGTCAATCGCTTCAAA
V Y K L G S I T R A
arfd1 (narD122) gtgacattttcagGTT TAC AAG CTG GGC TCA ATT ctA AGA GCG GTT GACGTCAATCGCTTCAAA
V Y K L G S I L R A
HindIII
arfd1(T653L) (oligo) gtgacattttcagGTT TAC AAG CT t GGC TCA ATT ctA AGA GCa GTT GACGTCAATCGCTTCAAA
V Y K L G S I L R A
arfd1T653L-1...gtgacattttcagGTT TAC AAG CTT GGC TCA ATT CTA AGA GCA GTT GACGT...
arfd1T653L-13...gtgacattttcagGTT TAC AAG CTG GGC TCA ATT CTA AGA GCA GTT GACGT...
sgRNA2
PAM
PpARFd1...ACA GGA CCA CAA CCT AAG ATT ACA CGG AGC TAC ATC AAGgtactttttctgagagtttgg...
T G P Q P K I T R S Y I K
NciI
arfd1(Y643*) (oligo) ACA GGA CCA CAA CCT AAG ATc ACc CGG AGt Tga ATt AAGgtactttttctgagagtttgg
T G P Q P K I T R S *
arfd1Y643*-4 ...ACA GGA CCA CAA CCT AAG ATC ACC CGG AGT TGA ATC AAGgtactttttctgagagtttgg...
arfd1Y643*-25 ...ACA GGA CCA CAA CCT AAG ATT ACA CGG AGT TGA ATT AAGgtactttttctgagagtttgg...
C
ARFd1-mYPet
Chloroplasts
arfd1T653L-mYPet #1
Chloroplasts
arfd1T653L-mYPet #13
Chloroplasts
D
Normalized mYPet Signal
1
2
3
4
*
*
ARFd1-mYPet /Δarfd2
arfd1T653L-mYPet /Δarfd2 #1
arfd1T653L-mYPet /Δarfd2 #13
