# Supplementary material for: Clade-D auxin response factors regulate auxin signaling and development in the moss Physcomitrium patens
Source: PLoS Biol. 2023 Jun 14;21(6):e3002163. doi: 10.1371/journal.pbio.3002163 (PMC10299833; doi:10.1371/journal.pbio.3002163)
Supplement: S5 Fig — (A) Complete table of values for arfd mutants grown on (+) or off (-) 5 μM IAA for 3 days. (B) Representative micrographs of protonemal filaments of 7-day-old regenerated protoplasts. White arrowhead indicates filament branch. Scale bar 50 μm. (C) Protonemal branching occurrence by cell position. Plants were scored after growing for 3 days on BCD, having regenerated from single protoplasts on PRMB for 4 days. There is a significant delay in the stabilized iaa2-mDII (n = 228 cells) line as well as a line lacking any F-box auxin co-receptor, Δafb1,2,3,4 (n = 144 cells) compared to wild type (n = 329 cells) (K.S. test, p < 0.001, 4 replicates for each line). (D) Complete table of values of data presented in (C). The underlying data for panels C and D are in S1 Data. (PDF) [file pbio.3002163.s005.pdf]

A

	*DR5:Ds*Red		*Δarfd1-18*		*Δarfd2-8*		*Δarfd^{dub}#1*		*Δarfd^{dub}#3*	
	-	+	-	+	-	+	-	+	-	+
Apical	0.0%	3.6%	0.0%	3.3%	0.0%	3.3%	0.0%	0.5%	0.0%	1.3%
1st Subapical	9.3%	39.6%	11.1%	37.4%	7.8%	26.8%	0.4%	7.5%	2.3%	12.3%
2nd Subapical	68.4%	50.9%	66.8%	52.0%	67.9%	59.2%	37.6%	60.4%	31.4%	60.2%
3rd Subapical	17.3%	4.1%	19.8%	5.7%	21.6%	9.2%	38.4%	23.5%	44.1%	22.0%
4th Subapical	3.7%	1.8%	2.0%	1.6%	2.4%	1.6%	15.6%	5.3%	15.1%	2.9%
5th+ Subapical	1.3%	0.0%	0.4%	0.0%	0.3%	0.0%	8.0%	2.7%	7.0%	1.3%

B

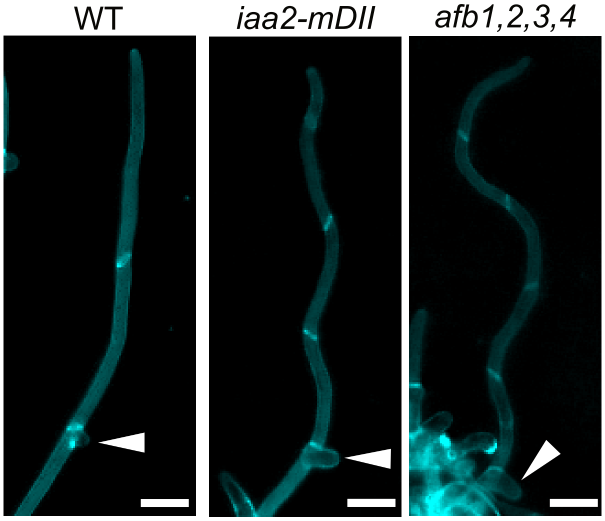

C

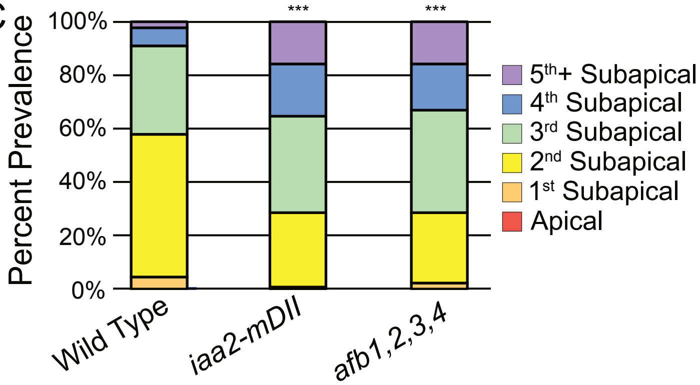

D

	Wild Type	*iaa2-mDII*	*afb1,2,3,4*
Apical	0.30%	0.00%	0.00%
1st Subapical	3.95%	0.88%	2.08%
2nd Subapical	53.50%	27.63%	26.39%
3rd Subapical	33.13%	35.96%	38.19%
4th Subapical	7.29%	20.18%	17.36%
5th+ Subapical	1.82%	15.35%	15.97%
