# Supplementary material for: Clade-D auxin response factors regulate auxin signaling and development in the moss Physcomitrium patens
Source: PLoS Biol. 2023 Jun 14;21(6):e3002163. doi: 10.1371/journal.pbio.3002163 (PMC10299833; doi:10.1371/journal.pbio.3002163)
Supplement: Attachment — Submitted filename: AuthorResponse_ARFd_v3.docx [file pbio.3002163.s008.docx]

**We thank the reviewers for the generally positive response to our manuscript but also the many helpful suggestions and critical comments. The reviewers' concerns are addressed as described below. We think the manuscript is greatly improved as a result.**

Rev. 1:
The manuscript by Bascom Jr. et al. presents a functional analysis of the D-class ARF proteins in Physcomitrium patens. It combines a genetic approach following a genetic screen for NAA-resistant (NAR) phenotypes, with phenotyping, expression and protein interaction analysis. It is neat and straightforward.

The authors identified in the screen three alleles in ARFd1 and constructed the other alleles by CRISPR, and the double mutant d1 d2 also by CRISPR.
Here I was confused with the labeling of the genotype in Fig S1 and Figs 2, 3, 4 (and others). In panel A of S1, are presented the gene deletion in arfd2-8, d2-8 d1-1 and d2-8 d1-3. But in panel B, the images are from d1 d2-1 and d1 d2-3. Can the authors checking the accuracy of the labeling in Fig S1 and 2? Maybe a table can be proposed to detail the genotypes used in the study?

**Thank you for bringing this to our attention. We generated both *arfd1,d2* mutants by knocking out *ARFd1* in the background of *arfd2-8*. We updated Fig S1 to make this point clearer. Additionally, we addressed this in the text.**

The authors analysed the effects of the mutations in the PB1 domain, the effects of NAA applications and high-nitrogen. They showed that there is an impact of the mutations of the timing of development of the branches. To further understand how D1 can act on activate transcription without having a DNA binding domain, the authors investigated its interaction with A-ARF and IAA1, by yeast two-hybrid and BCIF, proposing a model that D-ARF act as an enhancer of the activity of A-ARF on the promoters of auxin-responsive genes.

The authors indicated that D1 also interacts with A4, while only A8 is shown in Fig 7.

**Thank you for noting this error. We have omitted mention of ARFa4 from the text.**

I am also curious if the authors looked into the expression pattern (tissue and cell level) of the arfd1 T653L - mYpet. Is the mYPet protein expressed? Is there any differences [sic] with the WT D1-mYPet?

**We did look at localization and abundance of arfd1^T653L^mYPet. Curiously, we found that this protein was more abundant than the WT protein. We did not include these data in the original manuscript because we don't have an explanation for this observation and it is outside the scope of this study. However, at your request, we now include this data in Figure S3 and mention it in the text.**

These are only changes amendments. The manuscript is in state to be published when this is clarified.

Rev. 2:
In this manuscript, Bascom and colleagues address the function of Clade-D ARFs, a class of ARFs missing an obvious DNA-binding domain which is found only in bryophytes and lycophytes among extant land plants. To this end, they subject the Physcomitrium patens genes ARFd1 and ARFd2 to a functional characterization.
The manuscript is well written and to-the-point. Despite a relatively limited set of experiments and a compact format the authors show that clade-D ARFs act as regulators of physiologically significant auxin signaling in moss and also present a plausible model for their mode of action. Taken together, this is a major contribution of considerable news value with potential to attract the interest of colleagues studying plant development, land plant evolution and auxin function.

The authors should consider the following comments and suggestions to further strengthen the manuscript (ordered according to structure of manuscript rather than priority):

1. I have no questions, comments or suggestions relating to the title, abstract, or introduction*.*

2. Relating to Fig 1:
a. The authors could consider to remove narC18 and narD157 from the figure since the more severe phenotypes of these lines are not primarily related to mutations in ARFd genes.

**We agree with this suggestion. We have removed images of narC18 and narD157 from Figure 1 and simplified our discussion of these lines in the text.**

b. The phenotypic deviation in presence of NAA in narD122, nard122/Δarfd1, and Gd/NLS4Δarfd1 (marked by arrow heads) cannot really be seen. The authors should include inserts of gametophores at higher magnification.

**Thank you for pointing out this lack of clarity. We have included inserts that show a larger image of the relevant structures.**

c. Why does the DR5:DsRed2 output appear much stronger in Gd/NLS4Δarfd1 than narD122 and nard122/Δarfd1? Please comment. I suggest that also DR5:DsRed2 output in the absence of NAA is shown. Please add or explain why it is not needed.

**Although the *DR5:DsRed2* reporter was useful in the original screen, we have found expression of the reporter to be unstable. Sequence analysis revealed that the *DR5* reporter cassette was repeated ~ 60 times in tandem in this line. This is a possible explanation for the instability of the reporter. All other aspects of the mutant phenotype are stable.**

3. Relating to Fig S1:
a. The results section states that 'we used CRISPR/Cas9 to delete ARFd2 in the WT and Δarfd1 background', but when I look at Fig S1A I get the impression that ARFd1 was targeted in a pre-existing Δarfd2-8 background. Please change or clarify.

**You are correct. Both *arfd1,d2* lines were generated by deleting *ARFd1* in the *arfd2-8* background. The same *arfd2-8* background was used for the ARFd1mYPet lines**.

b. I would have liked to see the phenotypic characterization presented in Fig S1B-D and Fig 2 repeated for more independent single and double mutant lines. If such data exist, please include it. If not, I still find it acceptable in this particular case since additional and partly supporting phenotyping data is presented in other figures for other mutations in the same genes*.*

**While we generated multiple single mutants, we selected this subset to focus on for the paper. However, we now include additional data related to the single mutants, including gametophore counts (updated Figure 2C). In addition, we have repeated the branch timing assays for the single mutants and include this in an updated Figure 5H. We hope that the reviewer finds this acceptable**.

4. Relating to Fig 3:
a. In the results section discussing Fig 3b it is stated that 'arfd1T653L-mYpet lines exhibit an auxin sensitivity that is intermediate between the control and Δarfd1,d2 lines'. This is not very obvious from the data. Please provide statistical support for this claim.

**We appreciate your request for more statistical support. For Figures 2, 3, and 8 we conducted an ANOVA on the normalized chlorophyll content.**

b. Fig 3C indicates lower gametophore counts in arfd1Y543*-mYPet Δarfd2 lines than in the Δarfd1,d2 lines. Is there an explanation for this?

**We also noticed this effect but resisted the temptation to speculate upon it in the manuscript. One possible explanation is that *arfd1Y643*mYPet* has a weak dominant-negative effect by interacting with other proteins (co-activators) via the intrinsically disordered MR, thus reducing the pool available for interaction with wild-type ARFa/d proteins, or other TFs. Since we don't have any data that addresses this point, we prefer not to discuss it.**

5. Relating to Fig 4:
a. The finding that ARFds are required for caulonemal development only in nutrient-rich conditions is interesting. Can it imply that ARFd serves as a modifier of auxin responsiveness in response to nutrient availability? Maybe it could be worth to check if the expression of ARFd (and possibly auxin regulated genes likely to act downstream of ARFd) is regulated by the nitrogen source?

**Thank you for this interesting question. At your suggestion, we conducted qPCR on the *RSL6*, *IAA2*, and *ARFd1* genes with WT tissue grown on BCDAT or BCD (updated Figure S2). Interestingly, ARFd1 is not differentially expressed between the two tissue types.**

6. Relating to Fig 5 and the corresponding results section:
a. The data supporting that auxin affects the timing of branch initiation in Physcomitrium appears very solid. Still, after a quick look at Johri and Desai (1973) my impression is that their claim is that the proportion of caulonema-like branches is increased in response to auxin in Funaria, rather than the number of branches or the timing of branching. I may be wrong, but the author may want to comment on this.

**Thank you for pointing this out. We have clarified this in the discussion.**

b. The data presented appears to clearly support that branch initiation is delayed in the Δarfd1,d2 lines both in absence and presence of external auxin, but it is not as obvious if the ARFd genes are important for mediating the auxin-induced change in timing of branch initiation. The authors may want to provide statistical support for such a claim*.*

**As the reviewer states, our data clearly indicate that the *ARFd* genes contribute to the branch pattern. However, we now agree with the reviewer that the role of the *ARFds* in the auxin-induced changes in branch pattern is less clear. The additional statistical analysis that we performed was not conclusive. We speculate that this may be because of the complex nature of this developmental auxin response. In any case, we have removed the statement indicating that the *arfd^dub^* mutant is resistant to auxin with respect to branch patter.**

7. Relating to Fig 6:
a. The strong ARFd expression in protonemal cells at the base of gametophore initials is intriguing. An obvious question that comes to mind is if ARFd expression maxima in certain protonemal cells marks where branches differentiating into buds/gametophores will emerge. It is not a trivial experiment, but if the authors could provide data supporting such a function by some kind of time-lapse study it would be a major contribution.

**This is an excellent point and one we tried repeatedly to address. However, we found the technical challenges to be too great at this time and will defer this interesting question to a later date.**

8. Relating to Figure 7:
a. In the results it is stated that ARFd1 interacts with both ARFa4 and ARFa8, but I cannot find data supporting the former claim.

**Thank you pointing out this error. We have removed mention of ARFa4 from the text.**

b. In the results it is stated that ARFd1ΔDIV retains the ability to interact with ARFa8 but not with ARFb1, but I cannot find data supporting the latter claim.

**Thank you for pointing this out. We did not perform this experiment and have amended the text accordingly**

9. I find the discussion clear, relevant and to-the-point. I don't have any questions, comments or suggestions relating to the Material and Methods section.

*Rev. 3: Dolf Weijers – note that this reviewer has signed his review*
Bascom Jr and colleagues describe a genetic analysis of the Physcomitrium patens D-class ARFs. This is a mysterious subclade of the ARF transcription factor family (also referred to as non-canonical ARFs; ncARFs) that exist in bryophytes and lycophytes, but were lost in ferns and seed plants. A prior study in Marchantia showed that a D-class ARF mutant has reduced auxin response, but no detailed analysis was perfoemd, and no mechanism has been established. The authors of the present manuscript identified a D-class ARF mutant in a screen for auxin-resistant Physcomitrium mutants, and explore its role in auxin response. Overall, this reports important findings that are of importance for understanding the principles underlying auxin response. However, there are a number of points that require further exploration, clarification or elaboration*.*

1. The authors present a role of ARFds during P. patens development. However, unlike the authors suggest in the abstract, it does not seem to be "major role", given that this occurs only under specific nutritional conditions.

**We appreciate your point but argue that the defects exhibited by the *arfd^dub^* mutants are substantive and can be considered major. *Physcomitrium* growing outside of a growth chamber is likely to be in an environment containing nitrogen. However, clearly "major" is a subjective term and we have removed it from the title.**

2. While the "ARFd" nomenclature was coined by the authors (Lavy et al. 2016), the term D-class has not been widely used. Instead, ncARF (non-canonical ARF) has been used in the literature. I encourage the authors to explicitly state that this refers to the same proteins and propose the future used of only one name (ARFd)**.**

**We agree that this could be clearer and have amended the text accordingly.**

3. The interest in D-class mutations is clear, but the reader can be left wondering about the other mutations found in the mutagenized lines, as those in A and B-class ARFs, PINc or RSL7. I suggest to either leave out the information on the (putative) identity of these other mutants, or provide information on the molecular lesions, especially given the difference in the visible phenotypes in Fig.1 for narC18 and narD157.

**We agree that the presentation of the results of the screen was confusing. In the revised manuscript we simply mention that we recovered 3 lines with lesions in *ARFd1* and follow up with the genetic analysis of the locus in clean backgrounds. Images of *narC18* and *narC157* have been removed.**

4. Similarly, while the authors perform CRISPR against ARFd1 in the narD122 line, this line alone is not proven to be complemented with a ARFd1 WT copy, so other mutations, that most likely have occurred due to the technique used, may be important for auxin sensitivity (maybe epistatic to ARFd1?). What other mutations were found in narD122?

**We understand that the way we presented these experiments was confusing. We deleted *ARFd1* in the wild type line to confirm that the observed phenotype is due to a mutation in this gene. We also deleted *ARFd1* in *narD122* to determine if the mutation was acting in a dominant fashion. We have amended the text for clarity.**

5. The authors make single and double arfd mutants, but quickly jump to chlorophyll content analyses. Are these mutants similar or different to WT? how do they look in BCD and BCDAT with or without different concentrations of auxins? Some of this data is in supplemental material but is never described or discussed*.*

**The purpose of Fig 2 is to demonstrate that the ARFds function in auxin response as measured by chlorophyll retention and induction of auxin-regulated genes. Other aspects of the mutant phenotype are described in Fig 3 (gametophore development), Fig 4 (caulonemal development), and protonema branch formation.**

6. Image quality in Fig. 1 and 2 is poor. Please provide better pictures, maybe higher magnification would convey the phenotypes better. This would help the reader in following authors´ interpretation for auxin resistance.

**We agree quality of these images is poor. We have replaced the images in Figure 1 with higher resolution images. We also repeated the experiment described in Figure 2 and generated new and better images.**

7. Authors only show DR5:dsRED expression in response to NAA. In order to properly compare DR5:dsRED expression it would be important to see DR5:dsRED activity on medium without auxin, and ideally also with IAA (rather than NAA).

**Although the *DR5:DsRed2* reporter was useful in the original screen, we have found expression of the reporter to be unstable. Sequence analysis revealed that the *DR5* element was tandemly repeated ~ 60 times in this line. This is a possible explanation for the instability of the reporter. All other aspects of the mutant phenotype are stable.**  *8.* In some cases, the DR5 line shows a somewhat different behaviour in auxin response compared to a WT. For example, the DR5 strain shows a much smaller reduction in chlorophyll content (22% vs 36%) compared to the WT strain. The authors describe this as "similar", but this difference is striking and not trivial. Also, together with other findings, it seems that this background has inherent auxin insensitivity. Is this something common in Reute/Grandsen cross progenies? Why not making mutants in a non-DR5 background since the responsiveness seems to be dampened here?

**We elected to make the new mutants in the Reute strain because it is fertile whereas Gransden is not.** **Although there may be differences in auxin response between the two strains these tend to be small and variable. It is possible that this difference is related to the large number of additional ARF binding sites in the *DR5* line. We repeated the chlorophyll retention experiments in figure 2 with an additional replicate and found that the difference between Reute and the *DR5* line was small.**

9. In conjunction with the comment above, I have some issues with using chlorophyll content as a way of quantifying auxin response. The authors mention later that arfd1,2 mutations lead to delays in gametophore formation and protonema differentiation on BCDAT. Doesn't this fact introduce a bias in the chlorophyll analysis? Ater [sic] 2 weeks, the composition of tissues in the mutants is very different form wild-type (i.e. WT should have more gametophores, whereas the arfd1,2 mutant should have relatively more chloronema). These different tissues would contribute to the overall chlorophyll content per gram of tissue in a unique way. Are these stages comparable?

**We developed the chlorophyll retention assay as a convenient proxy for auxin response. As shown initially in Fig 2, treatment of wild type tissue with NAA clearly results in chlorophyll loss. We don't know the molecular basis for this response, although we speculate that it may be due to auxin-dependent gene expression related to photosynthesis. In any case, we argue that the lack of information on mechanism does not detract from the usefulness of the assay. There are many examples of growth or developmental hormone response assays in which molecular mechanism is unknown. To emphasize the fact that we are using chlorophyll retention as a proxy, we report the response of each line normalized to the mock-treated plants. We feel that presenting the data this way highlights the differences in auxin response between wild type and the mutants tested without suggesting that clade-D ARFs directly regulate chlorophyll biosynthesis.**

**With respect to the reviewer's suggestion that auxin resistance in the mutants is related to a reduction in gametophores compared to the wild type, this does not match the data. Figure 2C shows that on 5 μM NAA, the wild type has fewer gametophores than the mutants yet the mutants are highly resistant in the chlorophyll retention assay.**

10. It is unclear why the authors did not include the original arfd/nard or arfd2 single mutants in the gene expression analyses (Fig. 2C). These single mutants are disregarded also from Fig. 3 on. The authors indicate that ARFd1 has a larger contribution to the auxin response than ARFd2. But this is entirely bound to the first chlorophyll content analysis and single mutants are not checked for any other analysis. Is there any specific reason?

**We thank you for this good suggestion. We now include gametophore data in the updated Figure 2C for both single mutants. Additionally, we added data for both lines in the protonemal branch initiation data (Figure 5H).**

Also, why using such a long IAA treatment (4.5 hours) to measure immediate auxin responsive gene activation?

**We based our experimental design on previous auxin-induced gene expression studies which also used a ~5 hour IAA treatment and identified many well-known auxin responsive genes (Lavy et al., 2016). We have added this reference to the text. Still other reports of auxin-induced gene expression had far longer treatments of NAA, both 13 hours (Prigge et al., 2010b) and 15-day treatments of NAA (Plavskin et al., 2016).**

11. The results suggest that ARFd proteins are involved in altering growth patterns in response to changing nutrient conditions. To provide mechanistic understanding, it would be important to know if (and how) ARFd expression and protein levels are modulated by the nutrient contrasts used here.

**The reviewer touches on a very interesting question. In general, we think that studies of nutritional regulation of protonema development are outside the scope of our manuscript. However, we did ask if *ARFd1* expression is affected by ammonium in the medium and found that it was not (updated Supplemental Figure 2E).**  *12.* ARFd1-mYPet expression seems to be very similar between chloronema and caulonema tissues. Since ARFd1 appears to also have an impact on timing of branch formation, it would be interesting to know if ARFd1 expression levels are different in the apical and subapical cells, and how this correlates with branch formation.

**This is an excellent point and one we spent a lot of time investigating. While we expected ARFd1mYPet signal to be highest in the second subapical cell (the branching cell), this cell often had the lowest ARFd1mYPet signal. In any case, the differences we observed were not always statistically significant, and therefore we did not include these data in the manuscript.**

Authors also claim that YPet is not detected in mature gametophores, but the picture is not clearly showing that, especially at this magnification, since it could still be expression at the basal parts of the mature gametophore.

**This is an interesting point, and we thank the reviewer for making it. We have selected a different micrograph for Figure 6D. At a relatively young stage, though when individual phyllids are clearly visible, we do see ARFd1mYPet expression in the basal region of the gametophores. The text has been revised to reflect this new data.**

13. An open question is the molecular mechanism by which ARFd acts. One option is that it supports transcriptional activation by A-class ARFs, another is that is acts as an Aux/IAA scavenger (as proposed by Mutte et al, 2018). The authors clearly favor the former throughout the text, and do not comment on the possibility of the latter at all. I would encourage the authors to use e.g. transient gene expression assays in protoplasts to test these two hypotheses. Otherwise, the existence of a second hypothesis should be mentioned in the discussion, and the claim of co-activation activity toned down.

**We agree that we should have mentioned the scavenger model and now do so (and citing Mutte et al., 2018). At the same time, we have added additional data that supports the co-activator model (updated Figure 8). By replacing the MR of ARFd1 with the MR from ARFb2, we show that MR composition is important for ARFd1 function. Further, we show that expression of the ARFd1 PB1 domain alone does not restore normal auxin response to the *arfd^dub^* line. While the two models are not mutually exclusive, we think this new data strongly support the co-activator model.**

14. In page 20, the authors write: "This is the first evidence in any system that ARF oligomerization is important for activity.". This statement is not entirely correct. Kato et al 2020 showed that the MpARF1 PB1 domain is essential for function, and that it can be replaced by a heterologous oligomerization domain. I would suggest removing this statement.

**At the reviewer’s suggestion, we have removed this statement**.  *15*. At the end of page 21, the authors write: "…most models suggest that ARF-ARF interaction occurs primarily via the dimerization domain adjacent to the DBD, and only when the proteins are bound to DNA (Boer et al., 2014)". This is also not entirely correct, ARFs dimerize through their DBD also in the absence of DNA. Moreover, a recent preprint showed that the DBD and PB1 act cooperatively in promoting dimeric DNA binding, indicating that both domains contribute to dimer strength.

**We have removed this sentence**.  *16.* The text needs some refinement to improve clarity:

- In the introduction, the authors refer to the DNA binding sequence of ARFs. While the shown sequence is indeed an ARF binding motif, this not the only one. This may lead to confusion for a general audience. I suggest to reword to auxin responsive elements, but given these specific piece of data adds nothing to the rest of the work, the sentence could be deleted.

**We have removed the specific DNA sequence of the AuxREs from the text.**

*-* Authors introduce only A- and B-class at the beginning but then refer to C-class. This can also be wrongly interpreted*.*

**We updated the text to emphasize that phylogenetic clades are separate from specific ARF function. However, the groupings are useful when speaking generally, and so we opted to keep references to the different ARF clades in the text.**

*-* Mutant nomenclature is not clear. While the single alleles are named properly in Fig. S1 (as with Δarfd2-8), the double mutants obtained are also dashed in the text and figures (Δarfd1,d2-1). This dash is misleading, especially since it refers to the ARFd1 allele.

**For clarity, we updated Figure S1 and the text to emphasize that we knocked out *ARFd1* in the *arfd2-8* background. Instead of a dash to denote the two double mutant alleles, we amended the text and figures and now refer to them as *Δarfd^dub^#1* and *#3.***

*-* Authors indicate in page 12 the use of NAA for the screening, but later in page 13 explain why they use NAA instead of IAA. This should be explained at the very beginning.

**For the mutant screen, we took advantage of the fact that NAA results in the accumulation of a brown pigment. As such, NAA-resistant mutants are readily identifiable (since they will be green). We have included this remark in the first paragraph of the Results section**.

*-* On page 14 the authors mention Figure 3C, D but Fig. 3D does not exist (it refers to Fig. 3B, C).
- On page 13 the authors say that "Previous reports that auxin treatment results in down regulation... photosynthesis", please provide a reference.
- On page 14 the sentence "To gain insight...narD122" would read easier if the authors used the term "mutation" instead of "mutant".
- In Figure 4 it would be much easier to interpret results if the type of medium used to grow plants is mentioned IN the figure (BCD resp. BCDAT) instead of in the figure description.
- Figure 5 D, G and J contain the same data replicated. While I guess this done for clarity, please explain or indicate this in the captions for the sake of transparency.
- Unmerge K-S tests in Fig. 5G and 5H for each of the mutant lines. Each line seems to behave differently (in mock conditions).

**Each issue has been addressed as suggested by the reviewer. With respect to Figure 5, we updated the figure completely (updated Figure 5H), and hope that the reviewer finds the changes acceptable and helpful.**

*- In general, captions are quite uninformative and lack data for proper figure interpretation such as age of plants, time of treatments, etc. Statistical tests, number of samples, and replicates should be shown as well.*

**Each figure legend has been updated to include the requested information.**
